# A Case of Life-threatening Actinomyces turicensis Bacteremia

**DOI:** 10.7759/cureus.6761

**Published:** 2020-01-24

**Authors:** Tikal Kansara, Monil Majmundar, Rajkumar Doshi, Kuldeep Ghosh, Mohammad Saeed

**Affiliations:** 1 Internal Medicine, New York Medical College-Metropolitan Hospital Center, New York City, USA; 2 Internal Medicine, University of Nevada, Reno School of Medicine, Reno, USA

**Keywords:** actinomyces turicensis, bacteremia

## Abstract

*Actinomyces turicensis* (*A. turicensis*) are normal commensals of the oral, gut, vagina, and skin flora. Infection with these organisms is usually benign, and bacteremia is rare. Here, we describe a case of an otherwise healthy female patient presenting with renal calculi and life-threatening *A. turicensis* bacteremia. The patient did not have any risk factors for *A. turicensis* bacteremia. The patient developed multi-organ dysfunction syndrome and received a biodegradable right ureter stent. The patient improved with urosurgical intervention and appropriate antibiotic coverage.

## Introduction

*Actinomyces* are gram-positive, facultative anaerobic bacilli and are normal commensals of the oral, gut, vagina, and skin flora [1]. In the majority of cases, the infection with these organisms goes unnoticed [2]. This is mostly due to the natural immunity of the body as well as the organism being susceptible to commonly available antibiotics [1]. Bacteremia with these organisms is rare. Localized *Actinomyces* infection can mimic malignancy, nocardiasis or cold abscess [2]. The majority of *Actinomyces *bacteremia patients described in the literature had an abscess or severe infection, to begin with, or were immunocompromised [3]. There are no reported cases of *Actinomyces turicensis (A. turicensis)* bacteremia in patients without any predisposing risk factors. We discuss a case of *A. turicensis* bacteremia in an otherwise healthy patient.

## Case presentation

A 52-year-old woman presented with concerns of lower abdominal and flank pain for three days. She had associated loss of appetite, vomiting, nausea, and low-grade intermittent fever. She denied hematuria, dysuria, diarrhea, or trauma. She had no chronic medical illness. Four years ago, she had an emergency department (ED) visit for urinary tract infection and two years prior for vaginitis. She was allergic to penicillin which causes a rash. On physical examination, her blood pressure was 92/55 mmHg, she was drowsy, responding to deep pain stimulus, and occasionally responding to verbal commands with mild right lower abdominal tenderness.

**Figure 1:**
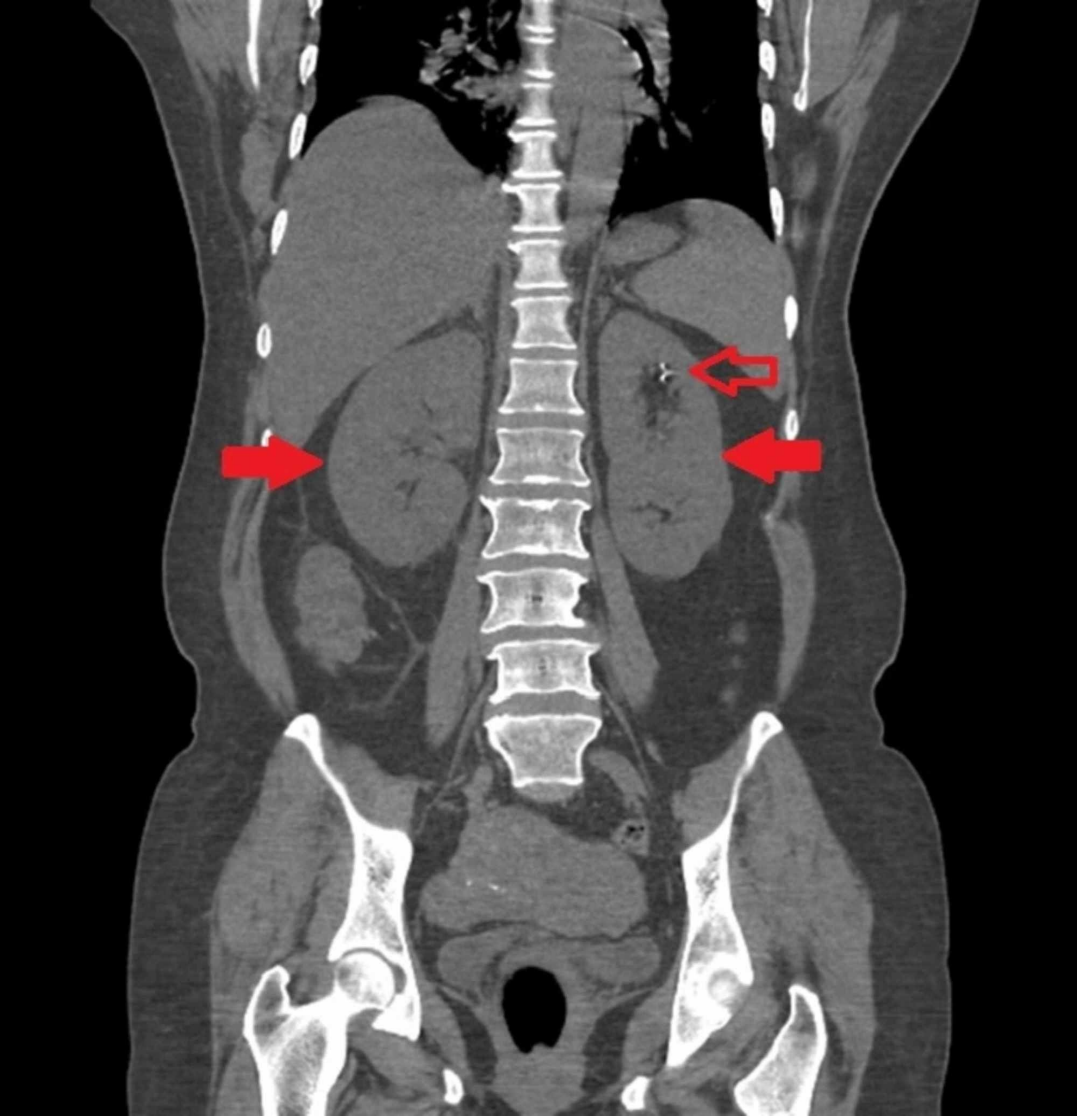
Computed tomography of the abdomen with contrast showing bilateral hydronephrosis (solid red arrows) with left non-obstructing renal calculi (red arrow)

**Figure 2:**
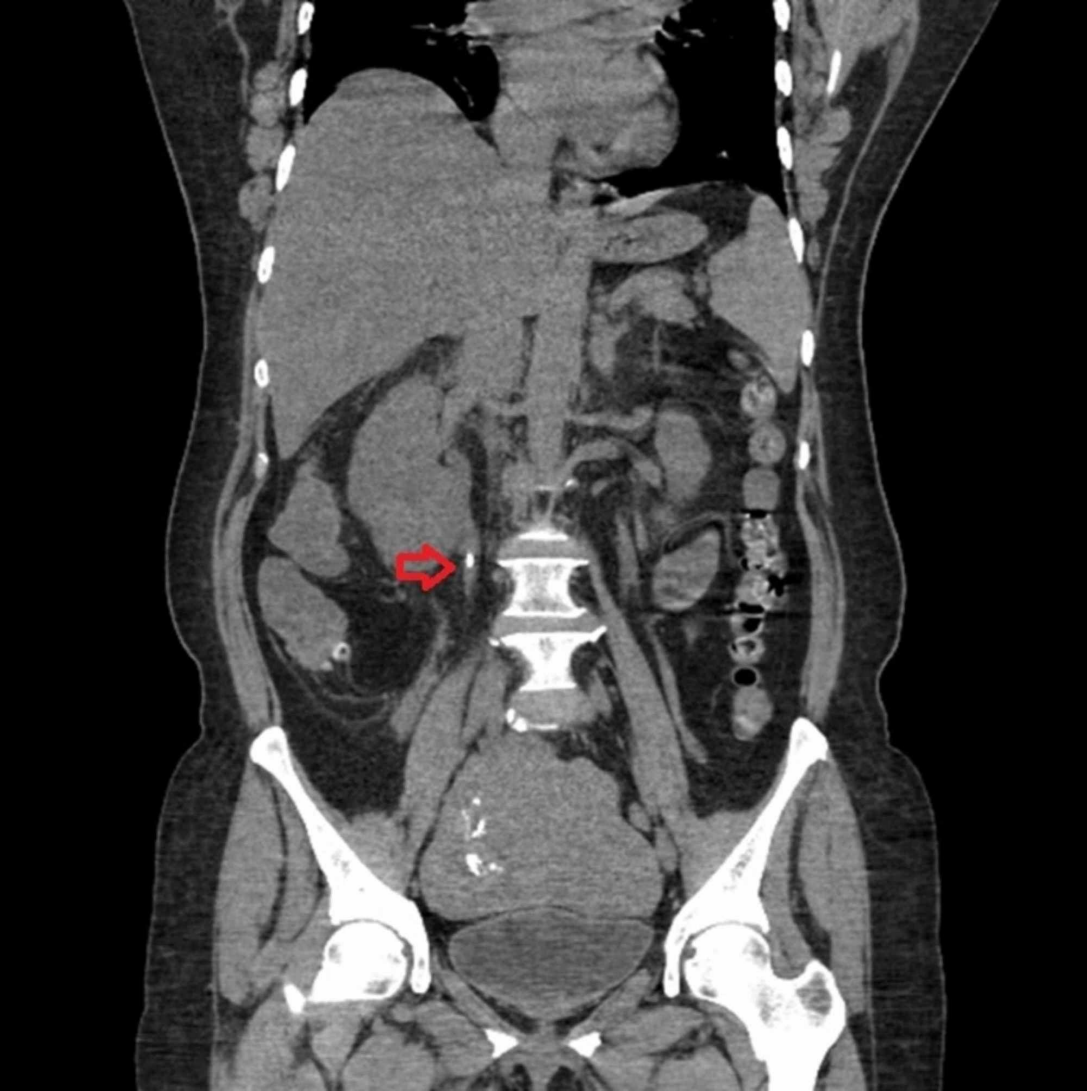
Computed tomography of the abdomen with contrast showing right-sided proximal ureteric renal calculi (red arrow)

**Figure 3:**
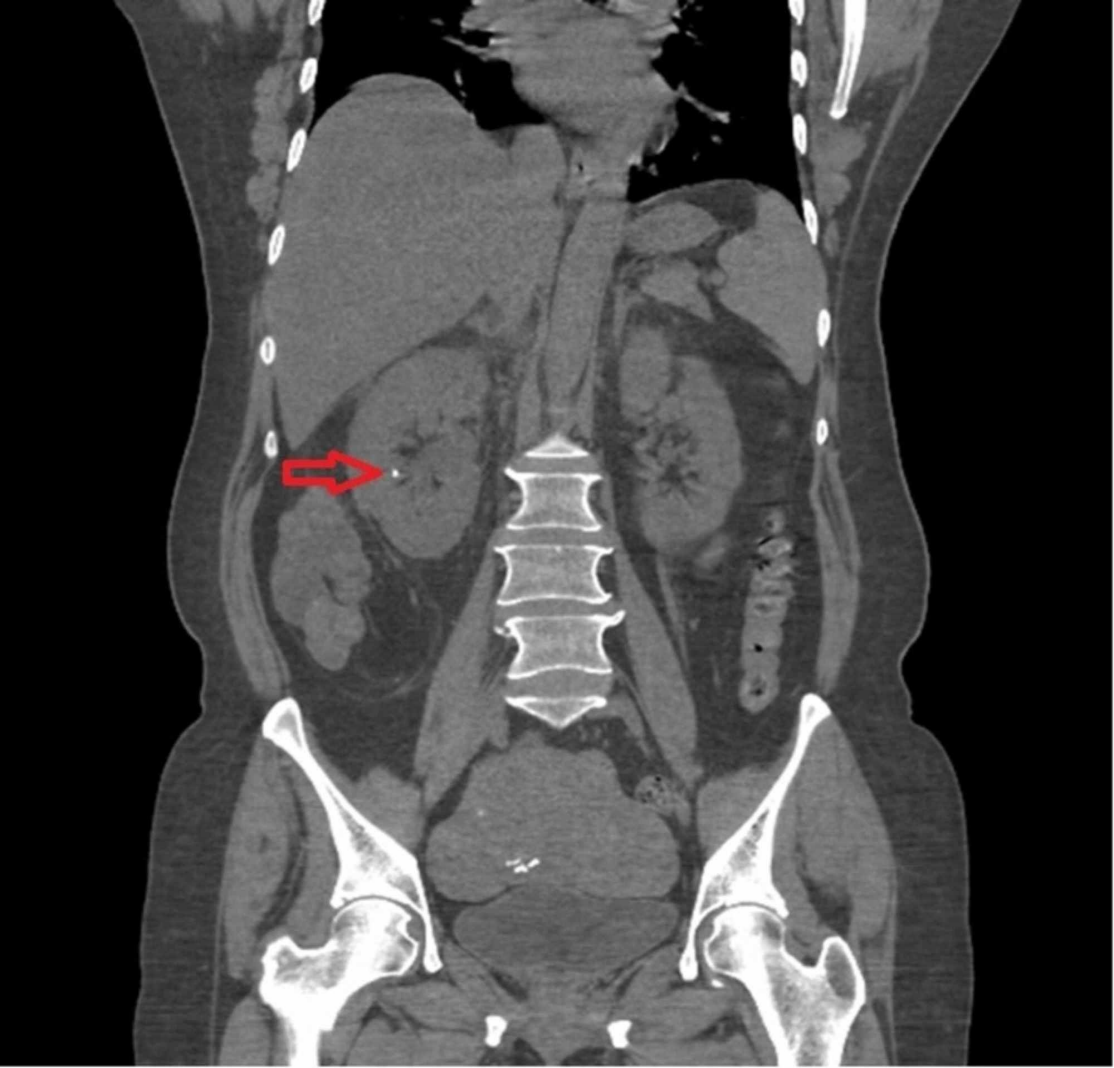
Computed tomography of the abdomen with contrast showing non-obstructive right renal calculi (red arrow)

Urinalysis showed 10 to 15 white blood cells (WBCs) per high-power field, moderate leucocyte esterase, and moderate blood in the urine. Complete blood counts revealed leukocytosis of 12.22k WBC/mcL (normal: 4.5k-11.0k WBC/mcL) with neutrophilia 88.1%, minimally elevated creatinine at 1.2 mg/dL (normal: 0.4-1.6 mg/dL) with a glomerular filtration rate of 47 mL/min/1.73 m^2^ (normal: > 60 mL/min/m^2^), and elevated transaminases with aspartate transaminase of 66 U/L (normal: 10-35 U/L) and alanine transaminase of 55 U/L (normal: 7-45 U/L). A computed tomography scan of the abdomen and pelvis showed right-sided hydronephrosis (Figure 1) and proximal hydroureter secondary to a linear stone measuring 6 mm in the proximal right ureter (Figure 2). Also, right-sided perinephric stranding and bilateral non-obstructing kidney stones-two on the right (Figure 3) and one on the left measuring 4 mm (Figure 1)-were noted. The uterus was enlarged with multiple calcified and non-calcified masses representing myoma. The results of other investigations including coagulation panel, hepatic panel, amylase, and lipase, were normal. Urine culture and two sets of blood cultures were drawn. She was given ceftriaxone empirically in the ED.

A diagnosis of septic shock with multi-organ dysfunction syndrome in the setting of urinary tract infection with impending pyelonephritis and abscess was made. The patient had to be admitted to the medical intensive care unit (MICU). She was started on intravenous vancomycin and meropenem and scheduled for ureteric stent placement. A right ureteric biodegradable stent was placed. A foley catheter was placed post-operatively to aid in recovery of left-sided hydronephrosis. Within the next few hours, the patient regained consciousness and was deemed stable. She was transferred out of the MICU, where she had hematuria and lower abdominal pain for two days, likely due to stent placement, which resolved completely. She was continued on vancomycin and meropenem for another nine days.

The blood cultures drawn on admission were found be positive for *A. turicensis* sensitive to penicillin, tetracycline, clindamycin, and ampicillin on the fourth day. Urine culture was negative for any growth. Based on culture and sensitivity, ceftriaxone was started, as the patient was allergic to penicillin, and continued for six days, for a 15-day regimen of antibiotics. The patient was discharged to home on 10th day with a regular follow-up in the clinic for general health care maintenance. She had no further symptoms. Her stent was removed in an outpatient setting. Culture of the removed stent did not grow *A. turicensis *organism.

## Discussion

The *Actinomyces* species are facultative anaerobic gram-positive bacilli with a filamentous appearance [1]. *Actinomyces* are common inhabitants of the gut and genitourinary tract [3]. In women, *Actinomyces* commensals include *A. meyeri, A. neuii, A. radingae, A. turicensis*, and *A. urogenitalis* [1,3-5]. The organism resides on mucosal surfaces and gains access to deeper tissues via trauma, surgical procedures, or foreign bodies, which disrupt the mucosal barrier [3]. Human actinomycosis is a chronic granulomatous infectious disease caused by *Actinomyces *species [3]. Typical actinomycosis is characterized, according to the body site, as orocervicofacial, thoracic, and abdominopelvic forms [6,7]. Abdominopelvic actinomycosis is usually a consequence of invasive procedures such as cholecystectomy and intrauterine contraceptive devices [7-11]. Abdominopelvic actinomycosis in women with *A. turicensis* is common in connection with urethritis, cystitis, adnexitis, endometritis, cervicitis, vaginitis, and vulvitis [12]. Our patient had urinary tract infection with septic shock, with two sets of blood cultures positive for *Actinomyces*, and a negative urine culture.

*Actinomyces* mostly grow in culture media admixed with other organisms and are, therefore, often overlooked [3]. There have been cases of actinomycosis abscess being misdiagnosed as tumors, only to later diagnose actinomycosis on histology of excised specimens [13]. On a thorough review of the literature, only a handful of cases of *Actinomyces* bacteremia were found. To our knowledge, this case is amongst the first 10 cases of *A. turicensis* bacteremia. Table 1 presents a summary of reported cases of *A. turicensis* bacteremia.

Summary of reported cases of *A. turicensis* bacteremia is as follows.

**Table 1:** All the reported cases of Actinomyces turicensis bacteremia

Year	No. of cases	Infectious source	Gender	Prognosis	Reference
1998	1	Unknown	Unknown	Unknown	Vandamme et al. [14]
1999	2	Skin abscess and sacral ulcer	Females	Survived	Sabbe et al. [12]
2001	3	Unknown	Unknown	Unknown	Hall et al. [15]
2002	1	Hepatic abscess	Male	Survived	Riegert-Johnson et al. [16]
2012	1	Pelvic abscess	Female	Survived	Ong et al. [17]
2015	1	Pyometra	Female	Survived	Hagiya [18]

Of the nine reported cases of *Actinomyces* bacteremia, four were in female patients, one in a male patient, and gender was not described in the remaining. The clinical signs are subtle, and this organism can be diagnosed only by culture or advanced techniques. In most cases, *Actinomyces* is difficult to detect as it grows mostly as mixed flora and is susceptible to common empiric antibiotics. Furthermore, it grows slowly on blood agar, takes seven days to grow at 35°C [12]. Histology of biopsied specimens, infected tissue, or pus is more sensitive than culture and can identify granulomatous inflammation, abscess, and necrosis along with sulfur granules in up to 75% of cases [18]. Immunofluorescence is highly specific for diagnosis. Molecular studies, such as 16S RNA sequencing, are a reference method for identification and classification. Polymerase chain reaction, with specific primers, could be used to identify microbial agents in clinical material [12,18]. None of these tests were performed for our patient because the organism was fully identified by blood cultures.

*Actinomyces* are generally susceptible to beta-lactam antibiotics and penicillin. Surgery and invasive procedures are considered in cases of extensive necrosis or failure of antibiotic therapy. Our patient was treated with vancomycin and meropenem initially until reports of culture were available, and then she was switched to ceftriaxone to complete 15 days of antibiotics. The patient improved significantly after the second day in the hospital, without any evidence of fever, hematuria, or abdominal pain which improved significantly after the second day of the hospital course.

## Conclusions

*A. turicensis* bacteremia is usually a benign infection with a good prognosis as the organism is mostly sensitive to routine antibiotics, including beta-lactams. However, in some cases such as our patient, the infection can lead to septic shock and rapid clinical deterioration if untreated. Even though literature describes infection with *Actinomyces* species in immunocompromised patients, it can sometimes occur in otherwise healthy individuals as in our patient. Larger studies are required to determine association of *A. turicensis* infection with renal calculi. Fortunately, once the appropriate antibiotic is given, complete recovery is possible in a relatively short term. 
